# A new species of *Scinax* from the Purus-Madeira interfluve, Brazilian Amazonia (Anura, Hylidae)

**DOI:** 10.3897/zookeys.706.14691

**Published:** 2017-10-04

**Authors:** Miquéias Ferrão, Jiří Moravec, Rafael de Fraga, Alexandre Pinheiro de Almeida, Igor Luis Kaefer, Albertina Pimentel Lima

**Affiliations:** 1 Programa de Pós-Graduação em Ecologia, Instituto Nacional de Pesquisas da Amazônia, Av. André Araújo, 2936, 69060-001, Manaus, Amazonas, Brazil; 2 Department of Zoology, National Museum, Cirkusová 1740, 19300, Prague 9, Czech Republic; 3 Programa de Pós-Graduação em Zoologia, Universidade Federal do Amazonas, Av. Rodrigo Octávio 6200, 69080-900, Manaus, Amazonas, Brazil; 4 Instituto de Ciências Biológicas, Universidade Federal do Amazonas, Av. Rodrigo Octávio 6200, 69080-900, Manaus, Amazonas, Brazil; 5 Coordenação de Biodiversidade - CBIO, Instituto Nacional de Pesquisas da Amazônia, Av. André Araújo, 2936, 69060-001, Manaus, Amazonas, Brazil

**Keywords:** Amazonian rainforest, Amazonas, anuran diversity, Brazil, Rondônia, *Scinax onca* sp. n.

## Abstract

A new tree frog species of the genus *Scinax* from the interfluve between the Purus and Madeira rivers, Brazilian Amazonia, is described and illustrated. The new species is diagnosed by medium body size, snout truncate in dorsal view, ulnar and tarsal tubercles absent, nuptial pads poorly developed, skin on dorsum shagreen, dorsum light brown with dark brown spots and markings, white groin with black spots, anterior and posterior surfaces of thighs black, and iris bright orange. The advertisement call consists of a single short note, with 16−18 pulses and dominant frequency at 1572−1594 Hz. Tadpoles are characterized by body ovoid in dorsal view and triangular in lateral view, tail higher than body, oral disc located anteroventrally and laterally emarginated, dorsum of body uniformly grey-brown with dark brown eye-snout stripe in preservative, fins translucent with small to large irregular diffuse dark brown spots.

## Introduction

With nearly 70 currently recognized species, the genus *Scinax* Wagler, 1830 represents one of the most species-rich hylid genera in the Neotropics. Nevertheless, an increasing rate of new *Scinax* species recognition in the few last years (e.g., Fouquet et al. 2007, Brusquetti et al. 2014; Sturaro and Peloso 2014; Araujo-Vieira et al. 2015; Araujo-Vieira et al. 2016; Juncá et al. 2015; Ferrão et al. 2016) indicates that our knowledge of the actual species diversity in this genus is still very incomplete. Similarly, despite an intensive research in the last decades (e.g. Faivovich 2002, Faivovich et al. 2005, Duellman et al. 2016) many questions concerning our knowledge of phylogenetic relationships of this and correlated genus remain still an object of discussion.

The *Hyla rubra* species group was first recognized by Leon (1969). Several years later, Fouquette and Delahoussaye (1977) resurrected the generic name *Ololygon* Fitzinger, 1843 (type species *Hyla strigilata* Spix, 1824) to harbour the members of the *H. rubra* group and delimited five other species groups. However, the correct generic name to these tree frogs is *Scinax* Wagler, 1830 (type species *Hyla aurata* Wied, 1821) as noted by Pombal and Gordo (1991). Consequently, Duellman and Wiens (1992) defined the genus *Scinax* based on external morphology of adults and tadpoles, osteology, and reproductive behaviour.

Faivovich (2002) tested monophyly of species groups traditionally recognized in *Scinax* and defined two main monophyla: the *S. ruber* Clade (comprising members of the *S. rostratus* and *S. ruber* species groups) and the *S. catharinae* Clade (involving members of the *S. catharinae* and *S. perpusillus* species groups). In following comprehensive review of the systematics of Hylidae, Faivovich et al. (2005) confirmed the monophyly of *S. ruber* and *S. catharinae* Clades and recovered *Hyla uruguaya* (Schmidt, 1944) in sister position to the *S. ruber* Clade (*H. uruguaya* was transferred to *Scinax* to avoid paraphyly of the genus). In the phylogeny proposed by Faivovich et al. (2005) the *S. ruber* Clade is composed by *S. rostratus* and *S. uruguayus* species groups plus species unassigned to any group and the *S. catharinae* Clade consists of *S. catharinae* and *S. perpusillus* species groups.

Recently, Duellman et al. (2016) revised the phylogeny of the family Hylidae and proposed three major changes in *Scinax* taxonomy: (i) resurrection of the genus *Ololygon* to harbour species of the former *Scinax catherinae* Clade, (ii) introduction of a new genus *Julianus* for members of the former *Scinax uruguayus* species group (sensu Faivovich et al. 2005), and (iii) restriction of the genus *Scinax* to the members of the former *S. ruber* Clade. As noted by Duellman et al. (2016), the separation of *Ololygon* and *Scinax* was evident in the cladistic analyses published by Faivovich (2002) and Faivovich et al. (2005). Moreover, Faivovich (2002) already stated that “*Scinax* could be partitioned at the level of the *catharinae* and *rubra* clades, and certainly there are names available for them. If desired, *Scinax* is available for the *rubra* clade, and the name *Ololygon* could be applied to the *catharinae* clade”. In this paper, we follow Duellman et al. (2016) whose analysis is based on presently widest dataset.

**Figure 1.**
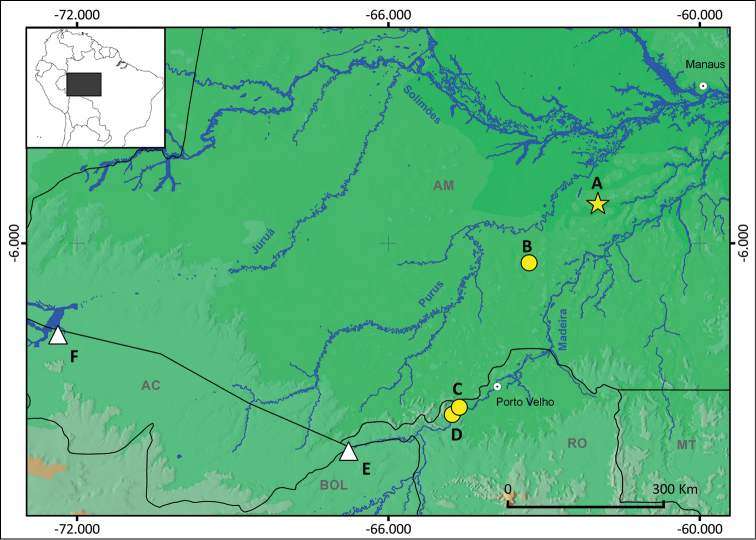
Distribution of *Scinax onca* sp. n. and *Scinax iquitorum* in Brazilian Amazonia. Yellow star: **A** type locality of *S. onca* sp. n., kilometre 350 of the BR-319 Highway, municipality of Beruri, State of Amazonas. Yellow circles: **B** paratype locality of *S. onca* sp. n., Floresta Estadual Tapauá Reserve, municipality of Tapauá, State of Amazonas **C–D** paratype localities of *S. onca* sp. n., municipality of Porto Velho, State of Rondônia. White triangles: **E** record of *S. iquitorum* near southern distribution of *S. onca* sp. n. according Melo-Sampaio and Souza (2015), municipality of Plácido de Castro, State of Acre, Brazil **F** record of *S. iquitorum* according Machado et al. (2015), municipality of Cruzeiro do Sul, State of Acre, Brazil.

The genus *Scinax* has a wide distribution area ranging from Mexico to central Argentina and Uruguay (Frost 2017). At present, 28 *Scinax* species are known to occur in Amazonia (see Sturaro and Peloso 2014, Brusquetti et al. 2014). However, a surprisingly high *Scinax* species diversity was recently revealed in the rainforests covering the area of the Purus-Madeira Interfluve (PMI; Fig. 1), where at least seven confirmed candidate species remain unnamed (Ferrão et al. 2016).

The PMI is crossed by an abandoned Trans-Amazonian highway (BR-319). Current proposals to reconstruct this highway bring a very serious threat for regional forest habitats and their fauna. Recent studies warn that one third of the PMI rainforest will be lost as a consequence of massive logging if this road improvement scheme goes ahead (Maldonado et al. 2012). Habitat loss has been widely reported as the major cause for populations decline and local extinctions for many groups of organisms, including frogs (e.g Soto-Azat et al. 2013). Therefore, current need of conservation of PMI is more urgent than ever before. In this respect, studies of species diversity resulting in descriptions of new species are of particular importance as they bring needed supporting data for wildlife conservation.

Here, we describe a new species of *Scinax* (*Scinax* sp. 3 *sensu* Ferrão et al. 2016) from the middle to southern PMI. The new species is described through external morphology of adults and tadpoles, and advertisement call.

## Materials and methods

Adult specimens of the new species were collected in four sampling areas in the PMI (Fig. 1). Two sampling areas are located in the middle portion of the study area, one at the kilometre 350 of the BR-319 highway (5°15'57"S, 61°55'58"W, ca. 59 m a.s.l.: Fig. 1A) and the other at the Floresta Estadual Tapauá Reserve (06°22'37"S, 63°17'19"W, ca. 69 m a.s.l.: Fig. 1B). The remaining two sampling areas are located in the southern region of the PMI, near the left margin of upper Madeira river, about 100 kilometres from the municipality of Porto Velho (9°9'32"S, 64°37'60"W, ca. 105 m a.s.l., Fig. 1C; 9°17'52"S, 64°46'10"W, *ca.* 101 m a.s.l., Fig. 1D).

All adult specimens were collected at night, anesthetised, and killed with topic solution of 10% benzocaine, fixed in 10% formaldehyde solution and stored in 70% ethanol. Tissue samples were obtained from all adult specimens and stored in 96% ethanol at Albertina Lima´s laboratory at INPA (Instituto Nacional de Pesquisas da Amazônia), Manaus, Brazil. Measurements were taken to the nearest 0.1 mm with digital calliper under a dissecting microscope. Sex and maturity of specimens were identified by observing secondary sexual characters (vocal sac, vocal slits), and gonads through dissection. The format for the description and diagnostic characters follows Duellman and Wiens (1993) and Duellman et al. (2006). Webbing formulae follow the standards of Savage and Heyer (1967) and Myers and Duellman (1982), while all other terminology is that of Duellman (1970), Heyer et al. (1990) and Napoli (2005). Measurement abbreviations used throughout the text are: SVL (snout–vent length), HL (head length, the straight line distance from the posterior edge of the jaw articulation to the tip of the snout), HW (head width at angle of jaw), IND (internarial distance), EN (eye to nostril distance), ED (horizontal eye diameter), ELW (upper eyelid width), IOD (minimal interorbital distance), TD (horizontal tympanum diameter), HAL (hand length), THL (thigh length) TL (tibia length), TAL (tarsus length), FL (foot length as the distance from the heel to the tip of the fourth toe), Fin3DW (Finger III disk width), Toe4DW (Toe IV disk width). Field notes and colour images were used for descriptions of coloration in life. Collected specimens were deposited in the herpetological section of the Zoological Collections of INPA (INPA-H). Specimens examined for comparative diagnoses are listed in the Appendix 1.

Tadpoles of the new species were collected in a 25m^2^ pond not connected to stream, in the sampling area near the kilometre 350 of the BR-319 highway (5°15'57"S, 61°55'58"W, ca. 59 m a.s.l.: Fig. 1A). Tadpoles were killed with a 5% lidocaine solution diluted in water, and preserved in 5% formalin (tail of one tadpole was stored in 100% ethanol). All tadpoles were deposited in one lot (INPA-H 35411) in the INPA-H collection. The determination of the tadpoles was verified using molecular barcoding (GenBank accession number KU317421; see Ferrão et al. [2016]). Tadpoles were staged according to Gosner (1960). The format for the tadpole description follows Schulze et al. (2015). The description was based on six tadpoles in the Gosner Stage 37. Following morphometric characters were measured according to Lavilla and Scrocchi (1986) and Altig and McDiarmid (1999): total length (TL), body length (BL), tail length (TAL), maximum width of the tail muscle (TMW), maximum height of the tail (MTH), maximum tail muscle height (TMH), interorbital distance (IOD), internarial distance (IND); eye diameter (ED) and eye-nostril distance (END).

The interspecific pairwise genetic distances in the 16S rRNA between the new species and other available *Scinax* species were presented by Ferrão et al. (2016). However, the intraspecific pairwise genetic distances between specimens of the new species remain unknown. Due to that, we calculated uncorrected p and Kimura-2-parameter distances (Kimura 1980) between 16S rRNA sequences from specimens of the two clades of the new species (see Ferrão et al. 2016, Fig. 2) with MEGA 6.06 (Tamura et al. 2013). Sequences of adults and one tadpole from middle PMI clade (KU317415, KU317416, KU317421, KU317422, KU317423, KU317425, KU317426) and of adults from southern PMI clade (KU317417, KU317418, KU317419, KU317420, KU317424, KU317427) were obtained from GenBank. Preliminarily to distance calculations, the sequence set was aligned using the Clustal W algorithm (Thompson et al. 1994) implemented in BioEdit (Hall 1999). Genetic distance is presented in the subsection “Variation”.

**Figure 2.**
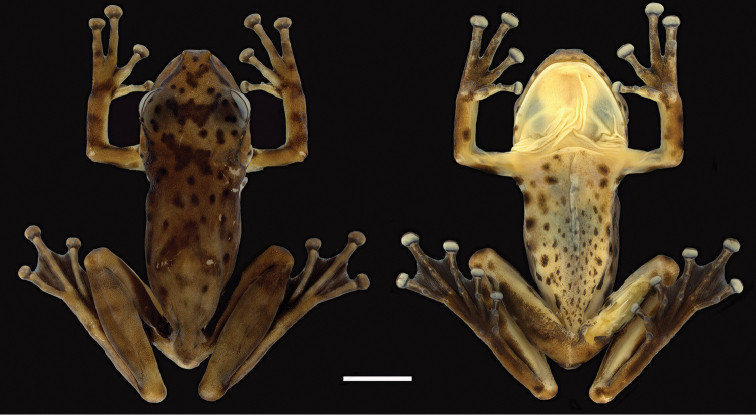
Holotype of *Scinax onca* sp. n. Dorsal and ventral view of the preserved holotype of *S. onca* sp. n. INPA-H 34584 from middle Purus-Madeira Interfluve, at the kilometre 350 of the BR-319 highway, municipality of Beruri, State of Amazonas, Brazil. Scale bar 5 mm.

Advertisement calls of one male (INPA-H 26624) from the Floresta Estadual Tapauá Reverve (middle PMI: Fig. 1B) were recorded in 12 October 2013 using a Sony PCM - D50 digital recorder. Air temperature at the time of recording was not measured. We analysed fifteen calls with the sound analysis software Raven 1.5 (Bioacoustics Research Program 2014). Obtained oscillograms and spectrograms were analysed through Blackman window, 80 Hz of frequency resolution and Fast Fourier Transformation (FFT) of 1024 points. The following call parameters were measured: call duration, inter-call interval, number of pulses per call, dominant frequency, and call repetition rate (number of calls emitted within one minute of vocalization). Terminology of call descriptions follows Köhler et al. (2017).

Institutional abbreviations are as follows:

**INPA-H** Collection of amphibians and reptiles of Instituto Nacional de Pesquisas da Amazônia, in Manaus, Brazil

**KU** University of Kansas, Museum of Natural History, Division of Herpetology, Lawrence, Kansas, USA

**QCAZ** Museo de Zoología, Pontifica Universidad Católica del Ecuador, Quito, Ecuador

**RMNH** Rijksmuseum van Natuurlijke Historie, Leiden, The Netherlands

**NHMG** Naturhistoriska Museet, Göteborg, Sweden

**NMP6V** National Museum, Prague, Czech Republic

**ZFMK** Zoologisches Forschungsinstitut und Museum Alexander Koenig, Bonn, Germany

## Taxonomy

### Scinax onca sp. n.

Taxon classificationAnimaliaAnuraHylidae

http://zoobank.org/5AF4775E-803F-4B1D-AAF6-6FFB94BDCD82

**Figure 3.**
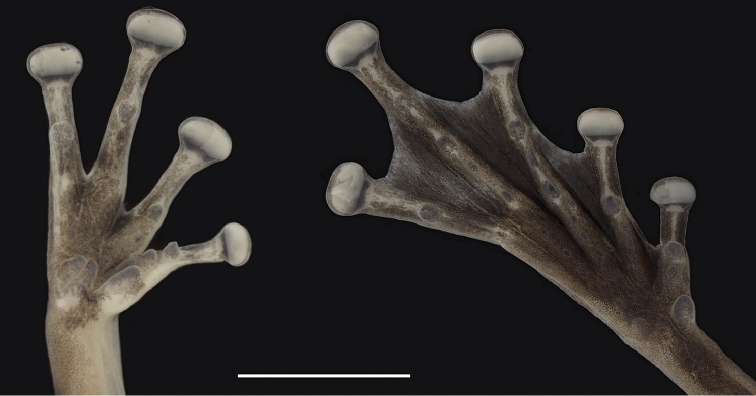
Hand and foot of holotype of *Scinax onca* sp. n. Ventral view of the hand and foot of the preserved holotype of *Scinax onca* sp. n. Scale bar 5 mm.

Figs 2 , 3 , 5 , 6 , 7 , 9 , 10 Suggested English name: Jaguar Snouted Treefrog.

Scinax iquitorum : Almeida et al. 2015: 142, Appendix II.
Scinax sp. 3: Ferrão et al. 2016: 7–9, figs 1 & 2B, Supporting table S2–S4.

#### Holotype

**Figure 4.**
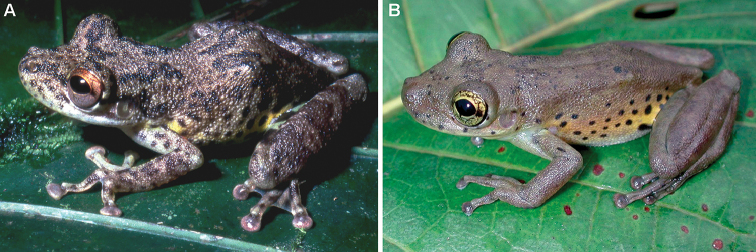
Adult specimens of *Scinax funereus* and *S. iquitorum*. **A** Female specimen of *Scinax funereus* (KU221960b) from San Jacinto, Region Loreto, Peru, and **B** male paratype of *Scinax iquitorum* (NMP6V 71267/1) from Puerto Almendras, Region Loreto, Peru. Photograph by W.E. Duellman (**A**) and Jiří Moravec (**B**).

(Figs 2–3, 4A–B). INPA-H 34584, an adult male from kilometre 350 of the BR-319 Highway (5°15'57"S, 61°55'58"W, ca. 59 m a.s.l., Fig. 1A), municipality of Beruri, State of Amazonas, Brazil, collected on 15 November 2013 by Miquéias Ferrão and Rafael de Fraga.

#### Paratypes

**Figure 5.**
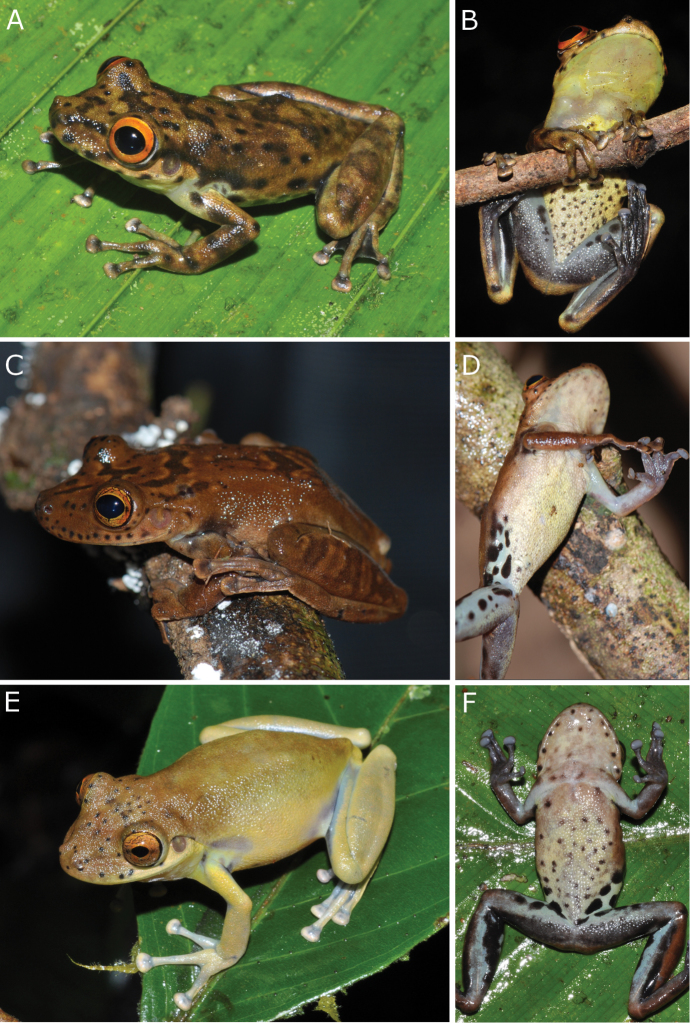
Colour in life of *Scinax onca* sp. n. Colour variation in life of *Scinax onca* sp. n. from the Purus-Madeira Interfluve, Brazilian Amazonia. **A–B** INPA-H 34584 (holotype), adult male from the kilometre 350 of the BR-319 highway, State of Amazonas **C–D** INPA-H 34591, adult female from municipality of Porto Velho, State of Rondônia **E–F** INPA-H 26625, adult female from the Floresta Estadual Tapauá Reserve, municipality of Tapauá, State of Amazonas. Photographs A–D and F were taken after transport of the specimens to the camp, while the image of E was taken immediately in the field.

**Figure 6.**
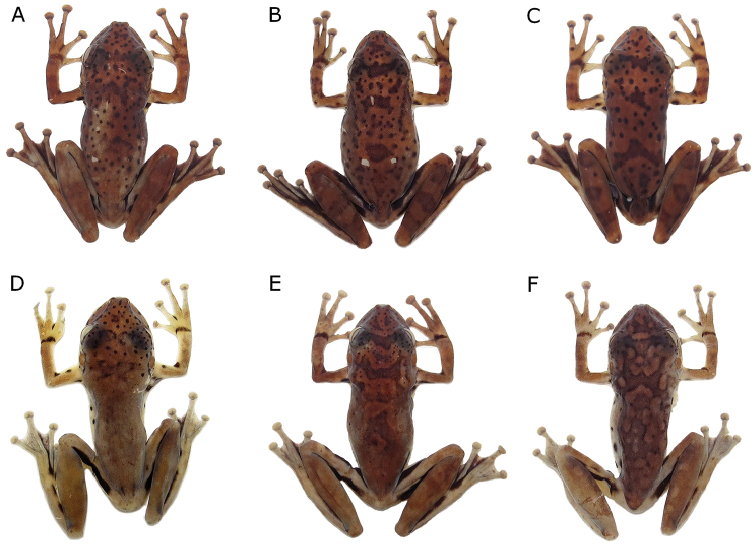
Colour in preservative of dorsum of *Scinax onca* sp. n. Dorsal colour variation of preserved specimens of *Scinax onca* sp. n. Specimens from middle (**A−C**) and southern (**D−F**) Purus-Madeira Interfluve, Brazilian Amazonia. **A** INPA-H 34581, male, SVL 34.3 mm **B** INPA-H 34583, female, SVL 35.5 mm **C** INPA-H 34582 male, SVL 31.5 mm **D** INPA-H 34594, male, SVL 32.6 mm **E** INPA-H 34589, female, SVL 38.9 mm **F** INPA-H 34593, male, SVL 34.5 mm.

**Figure 7.**
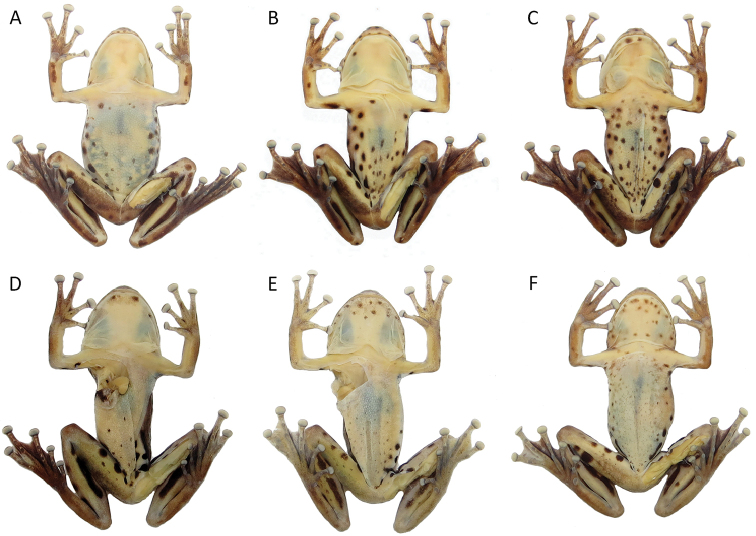
Colour in preservative of venter of *Scinax onca* sp. n. Ventral colour variation of preserved specimens of *Scinax onca* sp. n. Specimens from middle (**A−C**) and southern (**D−F**) Purus-Madeira Interfluve, Brazilian Amazonia. **A** INPA-H 34583, female, SVL 35.5 mm **B** INPA-H 34582, male, SVL 31.5 mm **C** INPA-H 34581 male, SVL 34.3 mm **D** INPA-H 34588, male, SVL 34.1 mm **E** INPA-H 34593, male, SVL 34.5 mm **F** INPA-H 34589, female, SVL 38.9 mm.

(Figs 5C–F, 6–7). Sixteen specimens: five adult males (INPA-H 34581, INPA-H 34582, INPA-H 34585, INPA-H 34586, INPA-H 34587) and one adult female (INPA-H 34583), same locality and collecting data as the holotype; one adult male (INPA-H 26624) and one adult female (INPA-H 26625) from the Floresta Estadual Tapauá Reserve (06°22'37"S, 63°17'19"W, ca. 69 m a.s.l., Fig. 1B), municipality of Tapauá, State of Amazonas, Brazil, collected on 12 October 2013 by Alexandre P. Almeida; five adult males (INPA-H 34588, INPA-H 34592, INPA-H 34593, INPA-H 34594, INPA-H 34595) and one adult female (INPA-H 34589) from municipality of Porto Velho (9°9'32"S, 64°37'60"W, ca. 105 m a.s.l., Fig. 1C), State of Rondônia, Brazil, collected on 2 November and 7 February 2014 by Albertina P. Lima; one adult male (INPA-H 34590) and one adult female (INPA-H 34591) from municipality of Porto Velho (9°17'52"S, 64°46'10"W, ca. 101 m a.s.l., Fig. 1D), State of Rondônia, Brazil, collected on 25 February 2010 by Albertina P. Lima.

#### Referred material.

Two: INPA-H 35413 and INPA-H 35414, newly metamorphosed specimens from the kilometre 350 of the BR-319 Highway (5°15'57"S, 61°55'58"W, ca. 59 m a.s.l., Fig. 1A), municipality of Beruri, State of Amazonas, Brazil, collected on 17 January 2014 by Miquéias Ferrão.

#### Generic placement.

We assign the new species to *Scinax* based on general morphological similarity to other members of the genus, cloacal tube of tadpoles positioned above the margin of the lower fin (a synapomorphy of the former *S. ruber* Clade sensu Faivovich [2002], currently *Scinax* sensu Duellman et al. [2016]).

#### Diagnosis.

A medium-sized species of *Scinax* characterized by the following combination of characters: (1) SVL 31.3−34.5 mm (n = 13) in males and 35.5−40.4 mm (n = 4) in females; (2) snout truncate in dorsal view, bluntly rounded in lateral view; (3) tarsal tubercles absent; (4) tubercles on lower jaw and knee absent; (5) skin on dorsum shagreen; (6) dentigerous processes of vomers triangular; (7) in life, ground colour of dorsum light brown with dark brown spots and markings; dorsolateral stripes or X-shaped blotch on dorsum absent; flanks light brown with or without dark brown spots; axillar region and groin white with black irregular spots; anterior and posterior surfaces of thighs black (usually bordered by an irregular white streak); webbing between toes black; belly white to yellow, with round dark brown spots; iris bright orange; (8) advertisement call consisting of a single pulsed note; note duration 102−121 ms; 16−18 pulses/note; dominant frequency 1572−1594 Hz; (9) tadpoles with body triangular in lateral view; labial tooth row formula 2(2)/3(1); labial arm absent.

#### Comparisons.

Until now, the following 28 valid species of *Scinax* occur in Amazonia (Sturaro and Peloso 2014, Brusquetti et al. 2014, Frost 2017): *S. baumgardneri* (Rivero, 1961), *S. blairi* (Fouquette & Pyburn, 1972), *S. boesemani* (Goin, 1966), *S. chiquitanus* (De la Riva, 1990), *S. cruentommus* (Duellman, 1972), *S. danae* (Duellman, 1986), *S. exiguus* (Duellman, 1986), *S. funereus* (Cope, 1874), *S. fuscomarginatus* (Lutz, 1925), *S. fuscovarius* (A. Lutz, 1925), *S. garbei* (Miranda-Ribeiro, 1926), *S. ictericus* Duellman & Wiens, 1993, *S. iquitorum* Moravec, Tuanama, Pérez & Lehr, 2009, *S. jolyi* Lescure & Marty, 2000, *S. karenanneae* (Pyburn, 1992), *S. kennedyi* (Pyburn, 1973), *S. lindsayi* Pyburn, 1992, *S. madeirae* (Bokermann, 1964), *S. nebulosus* (Spix, 1824), *S. oreites* Duellman & Wiens, 1993, *S. pedromedinae* (Henle, 1991), *S. proboscideus* (Brongersma, 1933), *S. rostratus* (Peters, 1863), *S. ruber* (Laurenti, 1768), *S. sateremawe* Sturaro & Peloso, 2014, *S. villasboasi* Brusquetti, Jansen, Barrio-Amorós, Segalla & Haddad, 2014, *S. wandae* (Pyburn & Fouquette, 1971), and *S. x-signatus* (Spix, 1824). Members of the genus *Julianus* occur in Uruguay, extreme southern Brazil, and in northern Corrientes, Argentina (*J. uruguayus* [Schmidt, 1944]) and in Serra do Cipó, Minas Gerais, Brazil (*J. pinimus* [Bokermann & Sazima, 1973]). Species of the genus *Ololygon* are distributed in Atlantic Coastal Forest of eastern Brazil, gallery forests of the Brazilian Cerrado and in Argentina (see Duellman et al. 2016). Among species of *Scinax* distributed in Amazonia, except by the species that occur in open habitats, all other species are endemic to the biome. Regarding the fact that *Scinax onca* sp. n. is an exclusive forest dweller known from the lowland rainforest of southern part of Central Amazonia we focus the comparison on Amazonian *Scinax* species, including six confirmed candidate species discovered recently in PMI (*Scinax* sp. 1–2 and *Scinax* sp. 4–7 of Ferrão et al. 2016).

Morphologically, *Scinax onca* sp. n. can be distinguished from all other Amazonian *Scinax* species by having bright orange iris and white groin with black spots in life and by the following combinations of characters (characters of other species in parentheses or brackets unless otherwise stated):

The new species differs from *S. baumgardneri*, *S. garbei*, *S. jolyi*, *S. kennedyi*, *S. nebulosus*, *S. pedromedinae*, *S. proboscideus*, and *S. rostratus* by snout truncate in dorsal view and bluntly rounded in lateral view, and by the absence of tubercles on the lower jaw and knee (elongated or pointed snout, and tubercles present on the lower jaw and knee; Duellman 1972, Pyburn 1973, Duellman and Wiens 1992, Lescure and Marty 2000, Lima et al. 2004). In addition, tadpoles of *S. onca* sp. n. differ from those of *S. garbei*, *S. nebulosus*, *S. pedromedinae*, and *S. rostratus* by the absence of labial arm (labial arm present; Duellman 1978, Hero and Mijares-Urrutia 1995, Duellman 2005, Gomes et al. 2014).

The male SVL 31.3−34.5 mm of *S. onca* sp. n. is larger than male SVL of *S. blairi* (27.8−30.1 mm; Fouquette and Pyburn 1972), *S. cruentommus* (24.8–27.7 mm; Duellman 1972), *S. danae* (24.5−27.4 mm; Duellman 1986), *S. exiguus* (18.0−20.8 mm; Duellman 1986), *S. fuscomarginatus* (15.7–26.7 mm; Brusquetti et al. 2014), *S. karenanneae* (SVL 26.6−28.9 mm; Pyburn 1993), *S. lindsayi* (about 24 mm; Pyburn 1992), *S. madeirae* (18.0–23.1 mm; Brusquetti et al. 2014), *S. villasboasi* (16.7–20.0 mm; Brusquetti et al. 2014), *S. wandae* (23.4–26.9 mm; Pyburn and Fouquette 1971), *Scinax* sp. 1 (20.2–22.5 mm, n = 5), *Scinax* sp. 2 (*sensu* Ferrão et al. 2016) (18.1–20.4 mm, n = 15), *Scinax* sp. 4 (*sensu* Ferrão et al. 2016) (23.2 mm), *Scinax* sp. 6 (*sensu* Ferrão et al. 2016) (25.1–26.7 mm, n = 6), and *Scinax* sp. 7 (*sensu* Ferrão et al. 2016) (22.6–25.9 mm, n = 28). The males of *S. onca* sp. n. are smaller than those of *S. fuscovarius* (SVL 41.0–44.0 mm; Cei 1980) and *S. sateremawe* (35.2–38.1 mm; Sturaro and Peloso 2014).

*Scinax onca* sp. n. can be distinguished from *S. boesemani* by conspicuous dark brown spots on the dorsum (light spots on dorsum) and belly (no spots), black posterior surfaces of thighs (light brown), and black webbing between toes (light brown; Goin 1966). The call of *S. onca* sp. n. differs from that of *S. boesemani* in duration (102–121 ms vs.160–290 ms in *S. boesemani*; Duellman and Pyles 1983).

The new species differs from *S. chiquitanus* in having snout truncate in dorsal view (rounded), head wider than body (narrower), black posterior surfaces of thighs (brown), and in having dark brown spots on the belly (light brown when present; De la Riva 1990). The call of *Scinax onca* sp. n. differs from the call of *S. chiquitanus* in duration (102–121 ms vs.185.3–338.8 ms in *S. chiquitanus*), number of pulses (16–18 vs. 23–42 in *S. chiquitanus*) and dominant frequency (1572–1594 Hz vs. 2100–2261.5 Hz in *S. chiquitanus*; De la Riva et al. 1994, Ferrão et al. 2016).

*Scinax onca* sp. n. differs from *S. ruber* by the snout truncate in dorsal view (rounded), black posterior surfaces of thighs (brown with yellow or orange mottling), and absence of dorsolateral stripes (tan to yellow dorsolateral stripes present; Duellman and Wiens 1993). There are seven available names in the synonymy of *S. ruber*: *Hyla conirostris* Peters, 1863 (type locality “Surinam”), *Hyla lateristriga* Spix, 1824 (type locality: Brazil, by implication), *Hyla lineomaculata* Werner, 1899 (type locality “Arima, Trinidad”), *Hyla robersimoni* Donoso-Barros, 1965 “1964” (type locality “Pajonales al sur de Macuro, Penisula de Paria, Venezuela”), *Hyla rubra hübneri* Melin, 1941 (type locality “Taracuá, Rio Uaupes”, “São Gabriel, Rio Negro”, and “Vicinity of Manaus”, all localities in the State of Amazonas, Brazil), *Scytopis alleni* Cope, 1870 (type locality State of Pará, Brazil, by lectotype designation of Duellman and Wiens 1993), and *Scytopis cryptanthus* Cope, 1874 (type locality “Nauta”, Region Loreto, Peru). According to their original descriptions, all these names are associated with specimens that have yellow blotches on the anterior and posterior surfaces of the thighs, and in some cases undersurfaces of tibiae (Moravec et al. 2009).

From *Scinax x-signatus* (Spix, 1824) the new species can be distinguished by absence of the X-shaped mark (present) and presence of dark brown spots on the dorsum (absent; Lutz 1973).

*Scinax onca* sp. n. differs from *S. ictericus* by snout truncate in dorsal view (bluntly round), absence of ulnar and tarsal tubercles (tubercles present), and by black posterior surfaces of thighs (light to dark brown; Duellman and Wiens 1993). The call of *Scinax onca* sp. n. differs from the call of *S. ictericus* in duration (102–121 ms vs. 70–90 ms in *S. ictericus*). The tadpoles of the new species differ in having triangular body in lateral view (ovoid; Duellman and Wiens 1993).

The new species can be distinguished from *S. funereus* (Fig. 4A) by its truncate snout in dorsal view (acutely rounded; Duellman 1978), absence of tarsal tubercles (a row of low tubercles on outer edge of tarsus; Duellman 1971), shagreen skin (strongly tuberculate; Duellman and Wiens 1993), flanks light brown (yellow; Duellman and Wiens 1993), orange iris (bicolored iris; see Fig. 4A), and black posterior surfaces of thighs (yellow with dark brown spots or pale with discrete dark brown blotches; Duellman and Wiens 1993). Labial tooth row formula 2(2)/3(1) of the tadpoles of *S. onca* sp. n. differs from that of *S. funereus* (2(2)/3; Duellman 1978). There are two available names in the synonymy of *S. funereus*: *Hyla depressiceps* Boulenger, 1882 (type locality “Ecuador”) and *Hyla rubra inconspicua* Melin, 1941 (type locality “Roque, Region San Martín, Peru”). According to the original description, *H. depressiceps* differs from the new taxon in having black and whitish marbled limbs. An examination of the holotype of *Hyla rubra inconspicua* shows that it differs by the presence of small tubercles on the head, dorsum and limbs including the tarsal area (see Moravec et al. 2009).

The new species differs from *S. iquitorum* (Fig. 4B) by snout truncate in dorsal view (bluntly rounded), dentigerous processes of vomers triangular (transverse), presence of conspicuous dark brown spots on dorsum (small dark brown dots concentrated only on head and in areas of scapular and sacral blotches), light brown flanks with or without dark brown spots (bright yellow flanks with numerous distinct round black spots), and by white long bones of hindlimbs (green; Moravec et al. 2009).

*Scinax onca* sp. n. differs from *Scinax* sp. 5 (*sensu* Ferrão et al. 2016) by light brown dorsum with dark brown spots (yellowish green with diminutive black spots), dark spots on belly (absent), and anterior and posterior surfaces of thighs black (uniformly yellowish green).

#### Description of the holotype.

Adult male 31.3 mm SVL. Body moderately slender; head wider than body, slightly longer than wide (HL/HW = 1.2, HL = 38.0% of SVL, HW = 32.3% of SVL); snout truncate in dorsal view, bluntly rounded in lateral view; nostrils markedly protuberant, elliptic, directed dorsolaterally; eye-nostril distance 76% of ED; internarial region moderately depressed; canthus rostralis rounded in both dorsal and lateral views; loreal region concave, more concave near to nostril; interorbital distance longer than upper eye width (IOD/ELW = 1.1), IOD 31% of HW; eye diameter 34% of HW; tympanic annulus distinct, tympanic membrane evident, rounded, 51% of ED; supratympanic fold present, slightly distinct; vocal sack subgular, bilobate; vocal slits extend from lateral base of tongue (slightly behind the half distance from the anterior edge) to the mouth angles; dentigerous processes of vomers triangular, bearing 7/6 (left/right) teeth; choanes rounded; tongue lanceolate.

Arm and forearm slender; axillary membrane absent; pectoral fold present; hand length 29% of SVL; fingers long bearing horizontally expanded discs; diameter of disc on finger III 49% of ED; relative length of fingers I<II<IV<III; palmar tubercle bifid, flat, longer than wide; thenar tubercle elongated; distal subarticular tubercle conical on Finger I, subconical on Finger II, rounded on fingers III–IV; supernumerary tubercles small, slightly distinct; nuptial pad poorly developed, slender, extending from proximal base of thenar tubercle to distal base of distal subarticular tubercle on Finger I; fingers II–IV basally webbed; fingers with narrow lateral fringes, external fringe on Finger IV extends to distal portion of thenar tubercle.

Hind limb long; tibia longer than femur, tibia length 52% of SVL, femur length 47% of SVL; tarsus length 27% of SVL; foot length 44% of SVL; toe discs more rounded than finger discs; diameter of disc on Finger IV 44% of eye ED; relative length of toes I<II<III<V<IV; inner metatarsal tubercle oval and flat; outer metatarsal tubercle rounded, flat, three times smaller than inner metatarsal tubercle; subarticular tubercles subconical on toes I–II, rounded on toes III–V; supernumerary tubercles small, rounded, and flat; webbing on toes I 2−2^+^ II 1^+^−2 III 1^+^−2 IV 2−1^+^ V; distinct external lateral fringe on Toe V extending to outer metatarsal tubercle; fringe on external margin of Toe I extends to inner metatarsal tubercle; tarsal folds and tarsal tubercles absent; tubercles on heels absent.

Skin on dorsum shagreen, almost granular in supratympanic and anterotympanic region; skin smooth on forelimbs, hind limbs, throat, chest, and vocal sac; skin areolate on belly and ventral surface of thighs.

#### Measurements of the holotype

**(in mm).** SVL 31.3; HL 11.9; HW 10.9; ED 3.7; EN 3.6; ELW 3.1; IND 2.8; IOD 3.4; TD 1.9; HAL 9.1; Fin3DW 1.8; TL 16.3; THL 14.8; TSL 8.6; FL 13.7; Toe4DW 1.6.

#### Colouration of the holotype in life

**(Fig. 5A–B).** Ground colour of dorsal surfaces of head, body, and limbs light brown; dorsal pattern consisting of W-shaped interorbital mark on the head, an irregular dark brown spot in scapular region, a Λ-shaped mark in sacral region, and numerous round dark brown spots distributed randomly on the head (including lips) and body; a conspicuous dark brown canthal stripe extends to tip of snout; a dark brown supratympanic stripe extends from corner of eye to anterior region of flanks; three dark brown transverse bars on the forearm, the proximal one extends to arm; three brown transverse bars on the tibia; fingers and toes light brown, distal surfaces of disc cream to tan, proximal surfaces grey; toe webbing black; axillar region white with small dark brown spots; flanks light brown with dark brown spots; groin white with dark brown spots; anterior surfaces of thighs black; posterior surfaces of thighs black, bordered with an irregular white streak; throat and vocal sac yellowish; chest translucent; belly yellowish laterally, white medially, covered with randomly distributed round dark brown spots; anterior ventral surfaces of thighs greyish with black spots; posterior ventral surfaces of thighs dark grey to black; ventral surfaces of hand and foot black; nuptial pad cream; iris bright orange, without black reticulation, bordered by black externally.

#### Colouration of the holotype in alcohol

**(Figs 2–3).** Dorsal surfaces of head, body, and limbs brown; throat, belly, and axillar area yellowish; groin white; dark brown dorsal and ventral pattern as in life with exception of inconspicuous transverse bars on thighs.

#### Variations.

Both uncorrected p and K2P distances between specimens from southern and specimens from middle PMI groups range between 0.4 and 1.1%. Both the p and K2P distances between individuals from middle PMI varied from 0% to 0.2% and between individuals from southern PMI varied from 0% to 0.6% (Table 1). Despite the high genetic similarity, it appears that some variation in measurements and coloration is evident between specimens from middle PMI and specimens from southern PMI (the straight distance between the closest localities is ca. 500 km).

**Table 1.** Intraspecific and interspecific genetic divergence. Uncorrected p-distance (upper-right) and K2P distance (lower-left) between 16S rRNA sequences of the paratype of *Scinax iquitorum* (1) and specimens of *S. onca* sp. n. from the southern (2–7) and middle (8–14) Purus-Madeira Interfluve. * = denote the sequence obtained from one tadpole of the lot INPA-H 35411.

	Specimens	1	2	3	4	5	6	7	8	9	10	11	12*	13	14
1	NMP6V 71267/3		0.059	0.063	0.059	0.059	0.057	0.059	0.059	0.059	0.059	0.061	0.059	0.059	0.059
2	INPA-H 34592	0.065		0.004	0.000	0.000	0.002	0.000	0.004	0.004	0.004	0.006	0.004	0.004	0.004
3	INPA-H 34588	0.070	0.004		0.004	0.004	0.006	0.004	0.008	0.008	0.008	0.011	0.008	0.008	0.008
4	INPA-H 34594	0.065	0.000	0.004		0.000	0.002	0.000	0.004	0.004	0.004	0.006	0.004	0.004	0.004
5	INPA-H 34593	0.065	0.000	0.004	0.000		0.002	0.000	0.004	0.004	0.004	0.006	0.004	0.004	0.004
6	INPA-H 34595	0.062	0.002	0.006	0.002	0.002		0.002	0.006	0.006	0.006	0.008	0.006	0.006	0.006
7	INPA-H 34589	0.065	0.000	0.004	0.000	0.000	0.002		0.004	0.004	0.004	0.006	0.004	0.004	0.004
8	INPA-H 20586	0.065	0.004	0.009	0.004	0.004	0.006	0.004		0.000	0.000	0.002	0.000	0.000	0.000
9	INPA-H 34585	0.065	0.004	0.009	0.004	0.004	0.006	0.004	0.000		0.000	0.002	0.000	0.000	0.000
10	INPA-H 34581	0.065	0.004	0.009	0.004	0.004	0.006	0.004	0.000	0.000		0.002	0.000	0.000	0.000
11	INPA-H 34583	0.068	0.006	0.011	0.006	0.006	0.009	0.006	0.002	0.002	0.002		0.002	0.002	0.002
12	INPA-H 35411*	0.065	0.004	0.009	0.004	0.004	0.006	0.004	0.000	0.000	0.000	0.002		0.000	0.000
13	INPA-H 35413	0.065	0.004	0.009	0.004	0.004	0.006	0.004	0.000	0.000	0.000	0.002	0.000		0.000
14	INPA-H 35414	0.065	0.004	0.009	0.004	0.004	0.006	0.004	0.000	0.000	0.000	0.002	0.000	0.000	

The specimens from southern PMI exhibit slightly larger average size (t = -3.1, df = 10.4, p = 0.009) and significantly lower values of nine following male body proportions: HL/SVL (t = 2.3, df = 10.9, p = 0.01), IND/SVL (t = 3.4, df = 10.8, p = 0.005), IOD/SVL (t = 3.2, df = 9.6, p = 0.009), HAL/SVL (t = 6.9, df = 8.5, p < 0.001), THL/SVL (t = 2.8, df = 11, p = 0.01), TL/SVL (t = 3.9, df = 8.8, p = 0.003), TAL/SVL (t = 2.6, df = 10.2, p = 0.02), FL/SVL (t = 5.1, df = 10.3, p = 0.0003), and X3FD/SVL (t = 2.9, df = 6.7, p = 0.02). Variation of measurements and body proportions of the type specimens is given in Table 2.

**Table 2.** Morphometric data (in mm) of *Scinax onca* sp. n. from the Purus-Madeira interfluve, Brazilian Amazonia. Means followed by standard deviation, and ranges in parentheses. For abbreviations, see Materials and methods.

	Middle Purus-Madeira interfluve		Southern Purus-Madeira interfluve	
	Males (n = 7)	Females (n = 2)	Males (n = 6)	Females (n = 2)
SVL	32 ± 1.1 (31.3−34.3)	36.5 ± 1.0 (35.5−37)	33.6 ± 0.7 (32.6−34.5)	39.6 ± 1 (38.9−40.4)
HL	12.1 ± 0.3 (11.8−12.6)	13.1 ± 0 (13.1−13.1)	12.3 ± 0.3 (12−12.7)	13.6 ± 0.1 (13.6−13.7)
HW	11.3 ± 0.4 (10.9−11.9)	12.3 ± 0.3 (12.1−12.6)	11.8 ± 0.3 (11.4−12.1)	13.2 ± 0.1 (13.1−13.3)
ED	3.7 ± 0.3 (3.5−4.2)	3.7 ± 0.3 (3.5−3.9)	3.6 ± 0.2 (3.3−3.9)	3.9 ± 0.1 (3.8−4)
TD	2.1 ± 0.2 (1.9−2.4)	2.3 ± 0.3 (2.1−2.4)	2.2 ± 0.1 (2−2.4)	2.3 ± 0.1 (2.3−2.4)
UEW	3.1 ± 0.2 (2.7−3.2)	3.1 ± 0.1 (3−3.2)	3.2 ± 0.2 (2.9−3.4)	3 ± 0.1 (2.9−3.1)
IOD	3.3 ± 0.2 (3.1−3.7)	3.7 ± 0.3 (3.5−3.9)	3.2 ± 0.2 (3−3.4)	3.8 ± 0.2 (3.7−4)
IND	2.7 ± 0.1 (2.6−2.8)	3.2 ± 0.1 (3.1−3.3)	2.7 ± 0.1 (2.7−2.8)	3 ± 0.1 (2.9−3.1)
TAL	9 ± 0.3 (8.6−9.6)	10 ± 0.3 (9.8−10.2)	9 ± 0.3 (8.7−9.3)	10.5 ± 0 (10.5−10.5)
FL	13.7 ± 0.4 (13.4−14.4)	15.7 ± 0.4 (15.4−16)	13.4 ± 0.4 (12.9−14.1)	16.5 ± 0.4 (16.2−16.7)
HAL	9.5 ± 0.5 (9.1−10.4)	10.9 ± 0 (10.9−10.9)	9.2 ± 0.2 (8.9−9.5)	11.4 ± 0.7 (11−11.9)
3FD	1.8 ± 0.1 (1.7−1.9)	2.2 ± 0 (2.2−2.2)	1.7 ± 0.2 (1.5−1.9)	2 ± 0.2 (1.8−2.2)
4TD	1.7 ± 0.1 (1.6−1.8)	2 ± 0.1 (1.9−2.1)	1.6 ± 0.2 (1.4−1.8)	1.9 ± 0.2 (1.8−2.1)
END	3.9 ± 0.2 (3.6−4.2)	3.9 ± 0.2 (3.6−4.2)	4 ± 0.2 (3.7−4.2)	4.4 ± 0.1 (4.4−4.5)
TL	17 ± 0.5 (16.3−17.6)	19.2 ± 0.6 (18.7−19.6)	17 ± 0.4 (16.5−17.7)	19.8 ± 0.5 (18.5−19.2)
THL	15.9 ± 0.6 (14.8−16.5)	18.2 ± 0.7 (17.8−18.7)	15.8 ± 0.7 (14.7−16.9)	18.2 ± 0.7 (17.8−18.7)
HL/SVL	0.38 ± 0.01 (0.37−0.39)	0.36 ± 0.01 (0.35−0.37)	0.37 ± 0.01 (0.36−0.38)	0.34 ± 0.01 (0.34−0.35)
HW/SVL	0.35 ± 0.01 (0.35−0.37)	0.35 ± 0.01 (0.35−0.37)	0.35 ± 0.01 (0.34−0.37)	0.33 ± 0.01 (0.33−0.34)
ED/SVL	0.12 ± 0.01 (0.11−0.13)	0.10 ± 0.01 (0.10−0.11)	0.11 ± 0.01 (0.10−0.12)	0.10 ± 0.01 (0.09−0.10)
TD/SVL	0.06 ± 0.01 (0.06−0.07)	0.06 ± 0.01 (0.06−0.07)	0.06 ± 0.01 (0.06−0.07)	0.06 ± 0 (0.06−0.06)
UEW/SVL	0.10 ± 0.01 (0.09−0.10)	0.09 ± 0.01 (0.08−0.09)	0.10 ± 0.01 (0.09−0.10)	0.08 ± 0.01 (0.07−0.08)
IOD/SVL	0.10 ± 0.01 (0.10−0.11)	0.10 ± 0.01 (0.09−0.11)	0.09 ± 0.01 (0.09−0.10)	0.10 ± 0.01 (0.09−0.10)
IND/SVL	0.09 ± 0.01 (0.08−0.09)	0.09 ± 0 (0.09−0.09)	0.08 ± 0 (0.08−0.08)	0.08 ± 0.01 (0.07−0.08)
TAL/SVL	0.28 ± 0.01 (0.27−0.30)	0.28 ± 0.01 (0.27−0.28)	0.27 ± 0.01 (0.26−0.28)	0.27 ± 0.01 (0.26−0.27)
FL/SVL	0.43 ± 0.01 (0.42−0.45)	0.43 ± 0 (0.43−0.43)	0.40 ± 0.01 (0.38−0.41)	0.42 ± 0.02 (0.40−0.43)
HAL/SVL	0.30 ± 0.01 (0.29−0.31)	0.30 ± 0.01 (0.29−0.31)	0.27 ± 0.01 (0.27−0.28)	0.29 ± 0.02 (0.27−0.31)
3FD/SVL	0.06 ± 0.01 (0.05−0.06)	0.06 ± 0 (0.06−0.06)	0.05 ± 0.01 (0.05−0.06)	0.05 ± 0.01 (0.05−0.06)
4TD/SVL	0.05 ± 0 (0.05−0.05)	0.06 ± 0.01 (0.05−0.06)	0.05 ± 0.01 (0.04−0.05)	0.05 ± 0.01 (0.04−0.05)
END/SVL	0.12 ± 0.01 (0.12−0.13)	0.12 ± 0 (0.12−0.12)	0.12 ± 0.01 (0.11−0.13)	0.11 ± 0 (0.11−0.11)
TL/SVL	0.53 ± 0.01 (0.51−0.55)	0.53 ± 0 (0.53−0.53)	0.51 ± 0.01 (0.49−0.51)	0.50 ± 0.03 (0.48−0.52)
THL/SVL	0.50 ± 0.02 (0.47−0.52)	0.50 ± 0.01 (0.50−0.51)	0.47 ± 0.01 (0.45−0.49)	0.47 ± 0.02 (0.46−0.49)

Colour change was observed after (Fig. 5A−D, F) and before (Fig. 5E) human manipulation of the specimens. After manipulation, general colouration of individuals became darker and spots and blotches became more conspicuous. In preservative, individuals from the middle PMI (Fig. 6A−C) had a larger number of dorsal spots and blotches in comparison to specimens from southern PMI (Fig. 6D−F). Regarding ventral coloration in preservative, individuals from the middle PMI (Fig. 7A−C) had a larger number of spots, which were concentrated on the belly. In the south, individuals had smaller ventral spots, and these were concentrated on the throat (Fig. 7D−F).

#### Vocalization.

**Figure 8.**
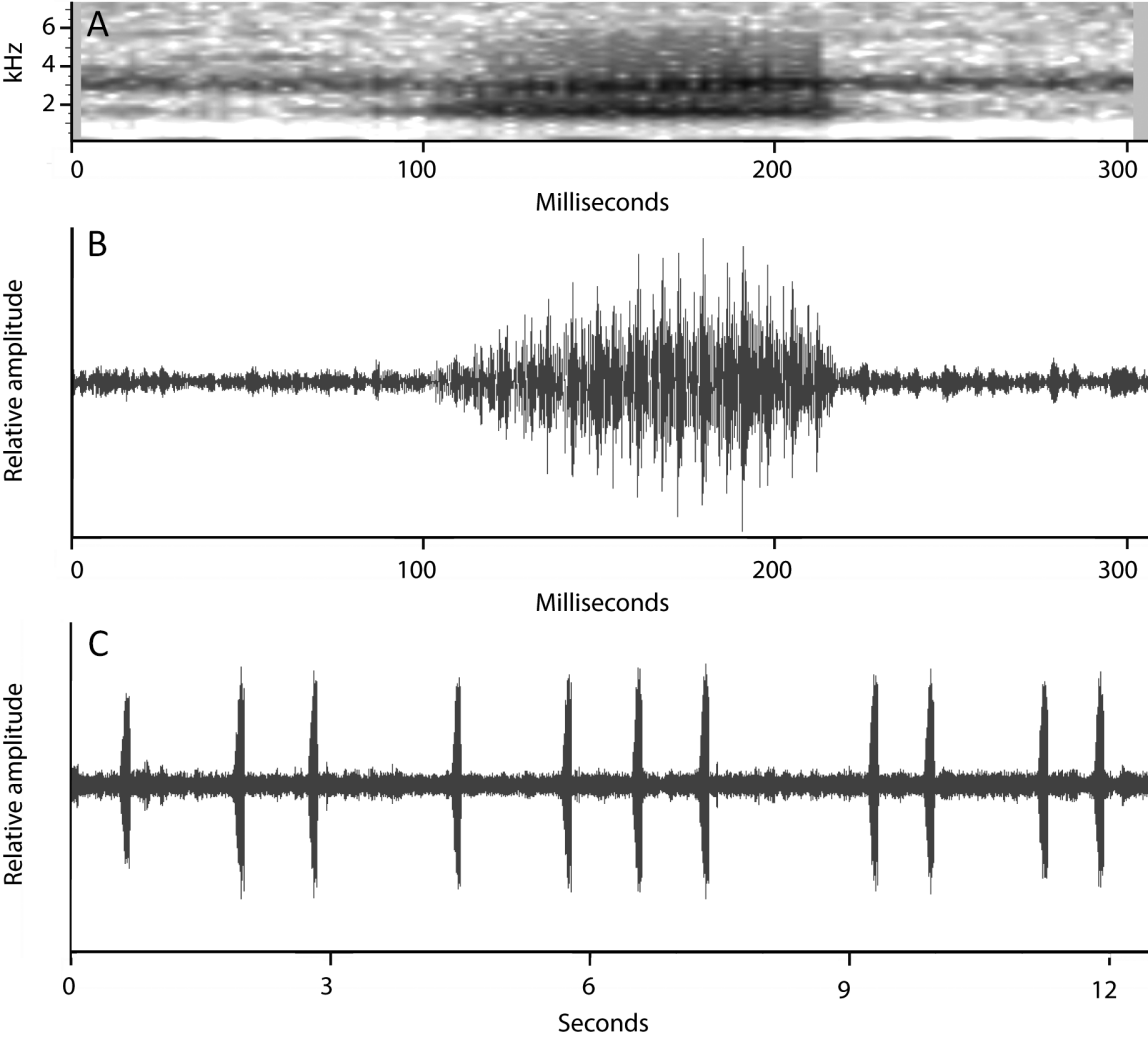
Advertisement call of *Scinax onca* sp. n. Spectrogram (**A**) and oscillogram (**B**) of an advertisement call of *Scinax onca* sp. n. The specimen (INPA-H 26624, SVL 32.1 mm) was recorded in Floresta Estadual Tapauá Reserve, middle Purus-Madeira Interfluve, Amazonas, Brazil **C** A series with eleven calls. Air temperature not measured.

The advertisement call of *Scinax onca* sp. n. consists of a single short multipulsed note (Fig. 8). Quantitative call parameters are as follows (range followed by mean ± standard deviation in parentheses): call duration, 102–121 ms (110 ± 5, n = 15); silent interval between calls 526–1844 ms (1089 ± 438, n = 15), pulses/call 16–18 (16.8 ± 0.8, n = 15); dominant frequency 1572−1594 Hz (1573 ± 6, n = 15). Calls were repeated at an approximate rate of 16 notes per minute.

#### Tadpole description.

**Figure 9.**
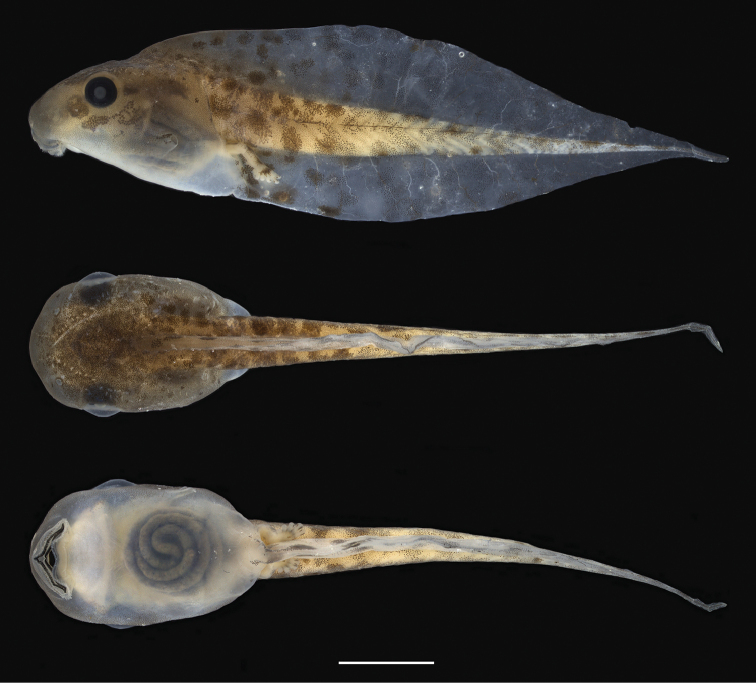
Tadpole of *Scinax onca* sp. n. from the middle Purus-Madeira Interfluve (lot INPA-H 35411). Specimen collected at kilometre 350 of the BR-319 highway, municipality of Beruri, State of Amazonas, Brazil. From top to bottom: dorsal, ventral, and lateral views of preserved tadpole in developmental Stage 37. Scale bar 5 mm.

**Figure 10.**
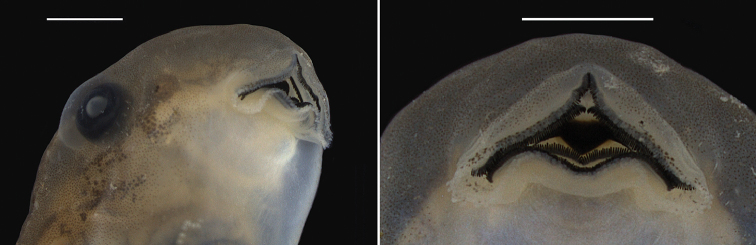
Oral disc of the tadpole of *Scinax onca* sp. n. (lot INPA-H 35411; developmental Stage 37). Left: ventrolateral and right: ventral view. The tadpole was collected in the middle Purus-Madeira Interfluve, at the kilometre 350 of the BR-319 highway, municipality of Beruri, State of Amazonas, Brazil. Scale bar 2 mm.

The following description is based on six tadpoles (Stage 37) of the lot INPA-H 35411. Total length 34.6−38.3 mm (37 ± 1.5, n = 6), body length 9.1−10.5 mm (9.8 ± 0.5, n = 6), and tail length 24.6−28.7 mm (27 ± 1.5, n = 5). Body ovoid in dorsal view, triangular in lateral view (Fig. 9). Snout rounded in dorsal and lateral view, distinct from body. Nostrils large, rounded, positioned and directed dorsally, eye-nostril distance represents 63−88% (74 ± 9, n = 6) of eye diameter. Inter nostril distance represents 62−70% (65 ± 3, n = 6) of inter orbital distance. Eyes large, positioned and directed laterally, with diameter 15−19 % (17 ± 1, n = 6) of body length. Spiracle tube single, sinistral, visible from dorsal view, inner wall and ventral right wall of the tube free from the body. Tail higher than body, point of maximum height of tail about half tail length. Tail musculature visible. Dorsal fin emerging nearly in the middle of the body, rising moderately, descending gradually to flagellum. Ventral fin approximately of the same height and shape as the dorsal fin. Cloacal tube positioned above the margin of the lower fin. Oral disc located anteroventrally, emarginated laterally, protuberant when closed (Fig. 10). Upper labium with uniseriate marginal papillae on distal portion and two rows of papillae (with small median gap) close to mouth angle. Lower labium with triseriate marginal papillae close to mouth angle and biseriate papillae on medial portion. Papillae are long, rounded on tip, distributed irregularly. Jaw sheaths moderately robust and serrated, upper jaw M-shaped and lower jaw V-shaped. Labial tooth row formula 2(2)/3(1). The row A-1 nearly the same length of A-2, P-2 slightly longer than P-1, P-3 shorter than P-1 and P-2. The gap in P-1 approximately the same length of the gap in A-2.

In life, dorsal and lateral surfaces of body silvery-green. Fins silvery-green, translucent, having dark grey spots. In preservative, dorsum of body uniformly grey-brown. A dark brown eye-snout stripe and dark brown interorbital blotch present. Fins translucent with small to large irregular diffuse dark brown spots. Tail musculature light brown. Ventral surfaces of the body white, slightly transparent.

#### Etymology.

The specific name *onca* refers to the Brazilian common name for the jaguar *Pantera onca* (Linnaeus, 1758) due the blotchy colour pattern of the new species. Furthermore, the specific name is a reference to frequent encounters of *P. onca* during the fieldwork in the PMI. The name is used as a noun in apposition.

#### Distribution, ecology, and threat status.

*Scinax onca* sp. n. is an exclusive forest dweller, known from two small areas located in the middle section of the PMI (State of Amazonas, Brazil), and two small areas lying in southern part of PMI, close to municipality of Porto Velho (Rondônia, Brazil). The maximum straight distance between the localities is around 500 km (Fig. 1). The middle PMI is covered by tropical lowland rainforest characterized by closed canopy with emergent trees whereas the southern part has a more open lowland rainforest formation with frequent palm trees.

The new species is an explosive breeder. All specimens were encountered after (or during) heavy rains when aggregated at middle-sized or large temporary forest ponds. The ponds were not connected to streams. The males were calling from shrubs growing in or next to the water. Calling males adopted both horizontal and vertical positions on leaves and shrub trunks ca. 50–200 cm above the ground. Other tree frogs found in sympatry with *S. onca* sp. n. included *Dendropsophus leucophyllatus* (Beireis, 1783), *D. marmoratus* (Laurenti, 1768), *D. minutus* (Peters, 1872), *D. parviceps* (Boulenger, 1882), *D. rhodopeplus* (Boulenger, 1882), *D. sarayacuensis* (Shreve, 1935), *Phyllomedusa vaillantii* Boulenger, 1882, and *Scinax* sp. 7 (*sensu* Ferrão et al. 2016).

Based on the sparse data available and due to threats, it is suggested that *S. onca* sp. n. be classified as “Data Deficient” according to the IUCN red list criteria (IUCN 2016). It is necessary to stress out, however, that the known range of the new species is seriously threatened by the planned reconstruction of the Trans-Amazonian highway BR-319 connecting Manaus and Porto Velho. This initiative will facilitate human migration from the “Arc of Deforestation” in southern Rondônia to the PMI (Fearnside and Graça 2006). According to recent predictions, this immigration could result in the deforestation of up 5.4 million hectares of mostly undisturbed rainforests between 2012 and 2050 (Maldonado et al. 2012). Three of four known *S. onca* sp. n. localities occur in the area of predicted deforestation. Only the fourth locality lies within the Floresta Tapauá Reserve, which can serve as refuge for this and other species.

## Discussion

The morphological data presented here show slight differences between members of the middle and southern PMI populations of *Scinax onca* sp. n. These differences are consistent with previously obtained molecular phylogeny (Ferrão et al. 2016), where the new taxon is structured into two slightly differentiated lineages corresponding to the middle and southern PMI populations. Nevertheless, until more robust evidence of specific distinction between the two populations is available, we decided to use the same specific epithet for the representatives of the both PMI populations. The observed differences may be a result of local adaptation to different environmental conditions, since the populations live under different climate conditions (drier in south) and inhabit different types of lowland forest. Other possibility is that the differences in measurements and coloration represent two examples of peripheral intraspecific variation, as the known populations are separated by ca. 500 km from each other. Till now we were not able to find individuals corresponding to *S. onca* sp. n. in the central region of the study area. Therefore, more complete sampling within this distributional gap is necessary to obtain a more exact picture of the morphological variation and genetic structure of *S. onca* sp. n.

Three records of *Scinax iquitorum*, species most closely related to *S. onca* sp. n., were recently reported from Brazilian western Amazonia (State of Acre; Machado et al. 2015, Melo-Sampaio and Souza 2015) and from Floresta Estadual Tapauá Reserve (State of Amazonas; Almeida et al. 2015). Two individuals reported by Almeida et al. (2015) were examined in this study. Since their morphology did not agree with the diagnosis of *S. iquitorum*, but corresponded fairly well with that of *Scinax onca* sp. n. (sensu Ferrão et al. 2016) from kilometre 350 of the BR-319 highway (type locality of *S. onca* sp. n., ca. 190 km from Floresta Estadual Tapauá Reserve) we included them into the type series of the new species described here. Individuals of *S. iquitorum* reported by Melo-Sampaio and Souza (2015) from the eastern corner of the Acre have very similar colour pattern to *S. onca* sp. n. from southern PMI, and we tentatively associate them with *S. onca* sp. n. Nevertheless, DNA barcoding as well as thorough morphological and bioacoustic data are necessary for a definitive determination of the specimens reported from eastern Acre. The same applies also for the proper determination of the individuals of *S. iquitorum* reported by Machado et al. (2015) from the municipality of Cruzeiro do Sul. In the light of contemporary knowledge of the extensive diversity of *Scinax* species in the State of Amazonas, we stress out that occurrence of *S. iquitorum* in the State of Acre should be verified by non-morphological traits.

It is evident that Brazilian States of Acre, Amazonas, and Rondônia house much more diverse fauna of *Scinax* tree frogs than previously thought. Similarly, this region is probably also home of many other, still unnamed, anuran species. Although a number of new species will be described in the near future, a more complete evaluation of the unique anuran diversity of the PMI is a long-term process, which is unlikely to be successfully completed without an effective wide-scale protection of the lowland Amazonian rainforest.

## Supplementary Material

XML Treatment for Scinax onca

## Appendix 1

List of specimens examined for morphological comparisons. Abbreviations: AM, state highway, State of Amazonas, Brazil; BR, Federal highway, Brazil; PDBFF, Biological Dynamics of Forest Fragments Project; UHE, Hydroelectric Power Plant.

*Scinax* sp. 1: BRAZIL: *AMAZONAS*: Tapauá, BR-319, km 450 (INPA-H 34688, INPA-H 34691, INPA-H 34689, INPA-H 34692, INPA-H 34690, INPA-H 34700).

*Scinax* sp. 2: BRAZIL: *AMAZONAS*: Humaitá, BR-319, km 620 (INPA-H 34651, INPA-H 34657, INPA-H 34664, INPA-H 34666, INPA-H 34667, INPA-H 34668, INPA-H 34669, INPA-H 34670, INPA-H 34671, INPA-H 34672, INPA-H 34673, INPA-H 34674, INPA-H 34675, INPA-H 34676, INPA-H 34677, INPA-H 34678).

*Scinax* sp. 4: BRAZIL: *AMAZONAS*: Humaitá, BR-319, km 620 (INPA-H 34693).

*Scinax* sp. 5: BRAZIL: *AMAZONAS*: Tapauá, BR-319, km 450 (INPA-H 34648, INPA-H 34656, INPA-H 34639, INPA-H 34640, INPA-H 34632); Berurí, BR-319, Km 260 (INPA-H 34703, INPA-H 34693, INPA-H 34696); Borba, BR-319, Km 220 (INPA-H 34710).

*Scinax* sp. 6: BRAZIL: *AMAZONAS*: Careiro da Várzea, BR-319, km 34, Ramal do Purupuru (INPA-H 34597); *RONDÔNIA*: Porto Velho, UHE Santo Antônio (INPA-H 35559, INPA-H 35561, INPA-H 35562, INPA-H 35563, INPA-H 35564, INPA-H 35565, INPA-H 35566, INPA-H 35567, INPA-H 35568).

*Scinax* sp. 7: BRAZIL: *AMAZONAS*: Careiro da Várzea, BR-319, km 100 (INPA-H 34600, INPA-H 34601, INPA-H 34604, INPA-H 34614, INPA-H 34615, INPA-H 34622, INPA-H 34598, INPA-H 34624, INPA-H 34627, INPA-H 34629), km 168 (INPA-H 34602); Borba, BR-319, km 220 (INPA-H 34610, INPA-H 34620); Beruri, BR-319, km 220 (INPA-H 34608), km 360 (INPA-H 34599, INPA-H 34607, INPA-H 34609, INPA-H 34611, INPA-H 34612, INPA-H 34617, INPA-H 34618, INPA-H 34621, INPA-H 34625, INPA-H 34626, INPA-H 34628, INPA-H 34630); Manicoré, BR-319, km 400 (INPA-H 34603, INPA-H 34606, INPA-H 34616, INPA-H 34623); Tapauá, BR-319, km 450 (INPA-H 34613, INPA-H 34619, INPA-H 34605, INPA-H 34665).

*Scinax boesemani*: SURINAME: *PARAMARIBO* (*SURINAME*): near Zanderij (RMNH12601, holotype). BRAZIL: *RORAIMA*: Caracaraí, Parque Nacional do Viruá (INPA-H 25972, INPA-H 25974).

*Scinax chiquitanus*: BRAZIL: *RONDÔNIA*: Porto Velho, UHE Santo Antônio, M-14 (INPA-H 35554, INPA-H 35555, INPA-H 35556, INPA-H 35557, INPA-H 35558, INPA-H 35560).

*Scinax cruentommus*: ECUADOR: *NAPO*: Santa Cecilia (KU 126587, holotype); *ORELLANA*: Parque Nacional Yasuní (QCAZ 8184), Río Napo (QCAZ 43772, QCAZ 44754). BRAZIL: *AMAZONAS*: Careiro da Várzea, BR-319, km 34, Ramal do Purupuru (INPA-H 34697).

*Scinax funereus*: ECUADOR: *ORELLANA*: Río Napo, Primavera (QCAZ 43799), Tambococha (QCAZ 55280, QCAZ 55283). PERU: *LORETO*: San Jacinto (KU221960b).

*Scinax fuscomarginatus*: BRAZIL: *RORAIMA*: Boa Vista, Estação Ecológica de Maracá (INPA-H 34662, INPA-H 34634, INPA-H 34646, INPA-H 34661); Caracaraí, Parque Nacional do Viruá (INPA-H 19371, INPA-H 19372, INPA-H 19376, INPA-H 19378, INPA-H 19383, INPA-H 19384).

*Scinax garbei*: BRAZIL: *RORAIMA*: Caracaraí, Parque Nacional do Viruá (INPA-H 25964, INPA-H 27496).

Scinax cf. ictericus: PERU: *MADRE DE DIOS*: Rio Tambopata (ZFMK 39353, ZFMK 39361, ZFMK 39363, ZFMK 39366).

*Scinax iquitorum*: PERU: *LORETO*: ca. 17 km straight SW of Iquitos, (NMP6V 71267/13; paratypes).

*Scinax madeirae*: BRAZIL: *RONDÔNIA*: Alta Floresta, Parque Estadual Corumbiaria (INPA-H 7050, INPA-H 7051).

*Scinax nebulosus*: BRAZIL: *PARÁ*: Alter do Chão (INPA-H 34647, INPA-H 34653); *RONDÔNIA*: Costa Marques, Real Forte Príncipe da Beira (INPA-H 34641); *RORAIMA*: Caracaraí, Parque Nacional do Viruá (INPA-H 27535, INPA-H 27536, INPA-H 27537).

*Scinax onca* sp. n. (tadpoles): BRAZIL: *AMAZONAS*: Beruri, BR-319, km 350 (lot INPA-H 35411).

*Scinax pedromedinae*: BOLIVIA: *BENI*: 5 km NE of Riberalta (NMP6V 70700); PERU: *UCAYALI*: Masisea (NMP6V 74902/1–3).

*Scinax proboscideus*: BRAZIL: *AMAZONAS*: Manaus, Reserva Colosso do PDBFF (INPA-H 10304); Presidente Figueiredo, Vila Pitinga (INPA-H 1870); *PARÁ*: Oriximiná, UHE Cachoeira Porteira, Rio Trombetas (INPA-H 304).

*Scinax sateremawe*: BRAZIL: *AMAZONAS*: Borba, Ramal Novo Horizonte (INPA-H 34695, INPA-H 34708).

*Scinax ruber* F: BRAZIL: *AMAZONAS*: Borba, BR-319, km 220 (INPA-H 34642, INPA-H 34652); *RONDÔNIA*: Porto Velho, UHE Santo Antônio, (INPA-H 34633, INPA-H 34635, INPA-H 34649, INPA-H 34655).

*Scinax ruber* PM: BRAZIL: *AMAZONAS*: Careiro da Várzea, AM-354, km 10 (INPA-H 34645, INPA-H 34654, INPA-H 34658, INPA-H 34659).
